# Supplementary material for: An open-source framework for large-scale, flexible evaluation of biomedical text mining systems
Source: J Biomed Discov Collab. 2008 Jan 29;3:1. doi: 10.1186/1747-5333-3-1 (PMC2276192; doi:10.1186/1747-5333-3-1)
Supplement: Additional file 1 — Evaluation platform source code. The additional file contains the software evaluation framework discussed in this paper. The most current release can be downloaded from SourceForge.net [1]. See the accompanying README file for instructions on installation and use. Also included in the distribution is a collection of UIMA wrappers for some commonly used BioNLP tools and annotated corpora. [file 1747-5333-3-1-S1.zip › bionlp_uima_repository-1.0.1/docs/api/edu/uchsc/ccp/util/external/geniatagger/GeniaTaggerOutputFileParser.html]

GeniaTaggerOutputFileParser (Center for Computational Pharmacology Util/UIMA API)


|  |  |  |
| --- | --- | --- |
| **Overview** \| **Package** \| **Class** \| **Tree** \| **Deprecated** \| **Index** \| **Help** | |  |
| PREV CLASS   NEXT CLASS | **FRAMES**    **NO FRAMES**     **All Classes** |  |
| SUMMARY: NESTED \| FIELD \| CONSTR \| METHOD | DETAIL: FIELD \| CONSTR \| METHOD |  |


---


## edu.uchsc.ccp.util.external.geniatagger Class GeniaTaggerOutputFileParser

```
java.lang.Object
  edu.uchsc.ccp.util.external.geniatagger.GeniaTaggerOutputFileParser
```

---

``` public class GeniaTaggerOutputFileParser extends Object ```

---

**Constructor Summary**

`GeniaTaggerOutputFileParser(String fileToParse)`


| **Method Summary** | |
| --- | --- |
| `boolean` | `hasNext(String documentText)` |
| `List<TextAnnotation>` | `next()` |
| `void` | `remove()` |

**Methods inherited from class java.lang.Object**

`clone, equals, finalize, getClass, hashCode, notify, notifyAll, toString, wait, wait, wait`

**Constructor Detail**

### GeniaTaggerOutputFileParser

```
public GeniaTaggerOutputFileParser(String fileToParse)
                            throws IOException
```

**Throws:**: `IOException`


**Method Detail**

### hasNext

```
public boolean hasNext(String documentText)
```

---


### next

```
public List<TextAnnotation> next()
```

---


### remove

```
public void remove()
```


---


|  |  |  |
| --- | --- | --- |
| **Overview** \| **Package** \| **Class** \| **Tree** \| **Deprecated** \| **Index** \| **Help** | |  |
| PREV CLASS   NEXT CLASS | **FRAMES**    **NO FRAMES**     **All Classes** |  |
| SUMMARY: NESTED \| FIELD \| CONSTR \| METHOD | DETAIL: FIELD \| CONSTR \| METHOD |  |


---
